# Monitoring soluble cMET and ctDNA in metastatic uveal melanoma patients to track early disease progression on immunotherapies

**DOI:** 10.1186/s13046-025-03451-2

**Published:** 2025-07-19

**Authors:** Devayani Machiraju, Christian H Ziener, Elena Clementi, Francisco García-Asencio, Jennifer Hüllein, Jasmin Richter, Bénédicte Lenoir, Melanie Wiecken, Daniel Hübschmann, Dirk Jäger, Jessica C Hassel

**Affiliations:** 1 Heidelberg University, Medical Faculty Heidelberg, Department of Dermatology and National Center for Tumor Diseases (NCT), NCT Heidelberg, a partnership between DKFZ and University Hospital Heidelberg, Heidelberg, Germany; 2 Heidelberg University, Faculty of Biosciences, Heidelberg, Germany; 3 Division of Radiology, German Cancer Research Center, Heidelberg, Germany; 4 Oncobit AG, Rütistrasse 16, Schlieren, Switzerland; 5 Computational Oncology Group (CO), Molecular Precision Oncology Program (MPOP), German Cancer Research Center (DKFZ); National Center for Tumor Diseases (NCT), NCT Heidelberg, a partnership between DKFZ and Heidelberg University Hospital, Heidelberg, Germany; 6 German Cancer Consortium (DKTK), Heidelberg, Germany; 7 Clinical Cooperation Unit Applied Tumor Immunity, National Center for Tumor Diseases (NCT) Heidelberg, German Cancer Research Center (DKFZ), Heidelberg, Germany; 8 Systems Immunology and Single-Cell Biology Group, German Cancer Research Center (DKFZ), Heidelberg, Germany; 9 Department of Dermatology, Venereology and Allergology, University Medical Center and Medical Faculty Mannheim, Heidelberg University, Mannheim, Germany; 10 Pattern Recognition and Digital Medicine Group, Heidelberg Institute for Stem Cell Technology and Experimental Medicine (HI-STEM gGmbH), Heidelberg, Germany; 11 Innovation and Service Unit for Bioinformatics and Precision Medicine, German Cancer Research Center (DKFZ), Heidelberg, Germany; 12 Institute of Human Genetics, Heidelberg University, Heidelberg, Germany; 13 Department of Medical Oncology, Heidelberg University Hospital, Heidelberg University, Heidelberg, Germany

**Keywords:** Metastatic uveal melanoma, Liver metastasis, Biomarkers, Immune checkpoint inhibitors, Tebentafusp, Liquid biopsy, Soluble cMET, Circulating tumor DNA

## Abstract

**Background:**

Metastatic uveal melanoma (mUM) is a rare malignancy and is different from metastatic cutaneous melanoma (mCM) in tumor characteristics and efficacy to immunotherapies. Tumor-specific biomarkers are required for mUM patients to monitor early disease progression on immunotherapies.

**Methods:**

We investigated clinical characteristics such as liver tumor burden and routine blood tumor markers, including lactate dehydrogenase (LDH) and transaminases in patients with mUM and with liver metastasized cutaneous melanoma (LmCM), treated with immune checkpoint inhibitors (ICIs) between May 2013-February 2024. In addition, we analyzed soluble cMET (scMET) in serum samples from these patients along with a cohort of mCM patients without liver metastases (nLmCM) using ELISA. Circulating tumor DNA (ctDNA) in the plasma was analyzed using digital droplet PCR (ddPCR) in mUM patients receiving immunotherapies. scMET, ctDNA, and LDH combination was used to simultaneously monitor disease progression in ICI and tebentafusp-receiving mUM patients.

**Results:**

Sixty-nine patients with mUM and seventy-six patients with LmCM were treated with either anti-PD1 monotherapy (*n* = 69, 48%) or ipi + nivo combination therapy (*n* = 76, 52%). Irrespective of the type of melanoma and type of immunotherapy, patients with liver metastasis size greater than 8cm experienced rapid disease progression. ICI-treated mUM patients with increased LDH, aspartate aminotransferase (AST), alanine transaminase (ALT), scMET, ctDNA, and rapidly growing tumors were significantly associated with treatment resistance and shorter progression-free and overall survival (*p* < 0.05). scMET (AUC: 0.82) outperforms LDH (AUC: 0.77) and S100 (0.68) in predicting one-year overall survival in these patients. A validation set with LmCM and nLmCM patient samples showed that increased scMET is likely a mUM-specific feature and does not predict ICI outcomes in LmCM or nLmCM patients (*p* > 0.05). Moreover, monitoring ctDNA and scMET in mUM patients under ICIs or tebentafusp treatment revealed the potential for early detection of disease progression.

**Conclusion:**

Soluble cMET might serve as a tumor-specific biomarker to predict clinical outcomes in mUM patients. A combinational assessment of scMET and ctDNA in mUM patients’ blood offers a highly sensitive potential approach to monitor early disease progression under immunotherapies with ICI or tebentafusp.

**Supplementary Information:**

The online version contains supplementary material available at 10.1186/s13046-025-03451-2.

## Introduction

Uveal melanoma (UM) is a rare form of cancer that is different from cutaneous melanoma (CM) in clinical and molecular characteristics [1–3]. In almost 50% of UM patients, the tumor spreads from the eye to distant organs [4]. This development is associated with grim overall survival (OS) of, on average, approximately one to two years [5–8]. Among the most prominent features of UM, its preferential metastasis to the liver and mutations in GNAQ or GNA11 genes in 90% of the patients are noteworthy.

In terms of systemic treatments for metastatic uveal melanoma (mUM) patients, immune checkpoint inhibitors such as anti-PD1 alone or combined with anti-CTLA4 and a soluble bi-specific TCR molecule called tebentafusp are currently being used. Immune checkpoint inhibitors (ICIs) have shown promising survival benefits in metastatic cutaneous melanoma (mCM) patients [9–12], and based on those findings, ICIs are currently being used as a standard of care for mUM patients. However, very few mUM patients show a favorable course of the disease upon ICIs compared to mCM patients, and biomarkers to identify these patients are limited [13, 14]. Meanwhile, tebentafusp has been the first systemic treatment shown to improve OS in HLA-A*02:01 positive mUM patients and is approved for treating HLA-A*02:01 positive mUM patients [7, 15]. Patients may benefit from tebentafusp even after the initial radiological appearance of disease progression [16]. These observations suggest that tebentafusp induces a change in the tumor microenvironment, which is profound enough to delay the rate of tumor progression to deliver a survival benefit but in a way that is not sensitively measurable by standard radiological techniques, highlighting the need for more sensitive biomarkers to monitor clinical outcomes.

UM spreads hematogenous; therefore, blood-based biomarkers can help monitor disease progression in these patients. Identifying biomarkers based on prominent features of UM, such as signaling pathways involved in liver metastasis and UM-specific mutations, can be promising. In this regard, expression of the Hepatocyte growth factor (HGF) receptor cMET on UM cells is known for its involvement in the preferential spread and growth of UM cells in the liver, as hepatic stellate cells produce HGF that serves as a cMET ligand and chemoattractant [17, 18]. Interestingly, a soluble form of cMET (scMET) is generated and released by the UM cells and can be seen in higher concentrations in serum samples of mUM patients [19]. Hence, we investigated if monitoring scMET concentrations might serve as a potential biomarker to predict immunotherapy outcomes in mUM patients.

Another promising blood-based biomarker is circulating tumor DNA (ctDNA). In UM, more than 90% of patients harbor mutually exclusive mutations affecting GNA11 and GNAQ genes. Although these mutations are not prognostic, they allow the detection of ctDNA in most patients using a targeted approach. The ctDNA level has been shown to correlate with LDH, tumor volume, PFS, and OS in mUM patients [20–22]. Furthermore, ctDNA rise preceded the radiological progression of the disease with a 4–10-week lead time [23]. ctDNA reduction in the early weeks on tebentafusp was associated with improved OS in patients in the phase III trial [24]. These observations imply that this test could be performed routinely as a complementary test to radiological scans and could serve as a regular monitoring modality for detecting early disease progression. However, there is limited knowledge of ctDNA detection for monitoring ICI response in mUM patients.

This study aimed to identify biomarkers to predict early disease progression in ICI and tebentafusp-treated mUM patients.

## Materials and methods

### Patient population and data sources

We performed a single-center, retrospective analysis of sixty-nine mUM patients who received ipilimumab plus nivolumab, nivolumab, or pembrolizumab between August 2014 and February 2024 (mUM ICI cohort, Additional Fig. 1a). Twenty-four mUM patients who received tebentafusp between July 2018 and September 2024, with blood samples available for scMET and ctDNA analysis, were analyzed (mUM tebentafusp cohort, Additional Fig. 1a). Patients received ICI or tebentafusp depending on the timing of tebentafusp's approval and their HLA status after approval. To determine the sensitivity of scMET, S100, and LDH markers collected simultaneously for predicting one-year overall survival, a cohort of 94 mUM patients was analyzed. Seventy-four patients in this cohort overlap with the mUM ICI and tebentafusp cohorts mentioned above. Additionally, twenty patients with available serum samples for scMET analysis were also included. Among these ninety-four patients, 40 received ICI treatment with ipilimumab plus nivolumab, nivolumab, or pembrolizumab, 20 received tebentafusp, and 25 received ICIs and tebentafusp sequentially. While three patients received ipilimumab, six patients did not receive any immunotherapies. Two hundred and sixty-two samples were measured for scMET from ninety-four mUM patients in total. To compare the potential biomarkers of mUM, information from a cutaneous melanoma cohort comprising 76 metastatic cutaneous melanoma patients with liver metastases (LmCM) and 227 patients with metastatic cutaneous melanoma without liver metastasis (nLmCM) at the time of ICI treatment and who received ipilimumab plus nivolumab, nivolumab, or pembrolizumab between May 2013 to August 2021 was retrieved (Additional Fig. 1b). Patient data was collected via the University Hospital Heidelberg electronic medical record system. Patient data was extracted manually. All clinical records were obtained with the approval of the Institutional Review Board (S-454/2015), and patients’ consents were obtained for scientific biobanking of peripheral blood (S-207/2005).

### Clinical data collection

Patient demographic information and tumor characteristics of mUM patients included age, sex, the time difference between stage I/II to stage IV diagnosis (time taken for stage IV development), systemic treatments before ICI or tebentafusp, and the presence of bone, liver, or brain metastasis at the start of immunotherapy (baseline) was retrieved from medical records. Hematologic markers collected included lactate dehydrogenase (LDH), C-reactive protein (CRP), alkaline phosphatase (ALP), aspartate transaminase (AST), alanine transaminase (ALT), S100, as well as absolute counts of lymphocytes, neutrophils, monocytes, eosinophils, and basophils at the time of ICI or tebentafsup treatment start and at all the corresponding times when scMET samples were collected.

### Clinical outcomes

Response to ICI was defined based on radiological evaluation according to Response Evaluation Criteria in Solid Tumours (RECIST) v1.1 criteria. Disease control (DC) was classified as those who derived clinical benefit from therapy, i.e., those who had complete response (CR), partial response (PR), or stable disease (SD). Non-responders were patients who had progressive disease (PD). Clinical endpoints included disease control rate (DCR), progression-free survival (PFS, *i.e.*, the time from the first dose of immunotherapy to progressive disease or death from any cause), and overall survival (OS, *i.e.*, the time from the first dose of immunotherapy to death).

### Radiological scans

Cross-sectional analysis of pre-treatment CT and MRI images that confirmed hepatic metastases in LmUM (*n* = 52) and LmCM (*n* = 69) patients were used for analysis. Dimensions of largest hepatic metastases and total liver diameter in CT or MRI images were taken manually with built-in measuring tools. Dimensions of the largest liver metastasis were categorized according to the AJCC classification for uveal melanoma in three groups for statistical analysis: tumor lesions > 3 cm (M1a), 3-8cm (M1b), and > 8 cm (M1c). In addition, the percentage of metastatic tumor volume in the liver was estimated and categorized into three groups: < 20%, 20–50%, and > 50% for statistical analysis.

### RNA sequencing in the NCT MASTER cohort

The NCT/DKTK MASTER (Molecularly Aided Stratification for Tumour Eradication Research) trial is a multicenter, prospective observational study based on comprehensive molecular diagnostics, therapeutic decision-making, and structured follow-up. The multi-omic workflow includes whole-genome sequencing (WGS) or whole-exome sequencing (WES) and RNA sequencing [25]. The MASTER cohort used in this analysis comprised forty-two patients enrolled in Heidelberg between September 2018 and December 2023, including 29 patients with mUM and 13 patients with mCM (Additional Fig. 1c). Before sequencing, all samples underwent quality control and estimation of tumor cell content by an experienced pathologist. Among the forty-two patients, transcriptome analysis couldn't be performed in two mUM patients and one mCM patient due to poor RNA quality in the tumor samples. Therefore, the transcriptomic analysis was evaluable only in 27 mUM patients and 12 mCM patients. Evaluation of TMB and RNA-seq was performed as previously reported [25]. Gene-level normalized RNA-seq data [tpm (transcripts per million) values] were used. MASTER study received Institutional Review Board approval from the Ethics Committee (Medical Faculty of Heidelberg University, reference number: S-206/2011.

### Serum and plasma collection

The pre-treatment samples were collected shortly before ICI treatment started, mostly on the day of treatment in the mUM or mCM patient cohorts and similarly for tebentafusp treatment in the mUM cohort. For the on-treatment samples in both mUM cohorts, samples were collected at staging, meaning about every 3 months. For the ICI cohort, an additional time point was taken at 6 weeks of treatment, and samples were collected before the third cycle. Further sequential samples were taken in the clinical routine; therefore, we have presented this information in the respective graphs in the results section of the manuscript. The number of patient samples in each cohort is presented in Additional Fig. 1a, b. Pre- and on-treatment peripheral blood samples were collected in serum tubes or EDTA-coated blood collection tubes and processed according to the standard NCT biobank protocols. The blood samples were centrifuged at 2,500 × g for 10 min for serum and plasma separation, divided into 200–300 uL aliquots, and stored at –80°C.

### ELISA

Serum concentrations of soluble cMET (ab277722, Abcam) were measured according to the manufacturer’s instructions. The intra- and inter-assay coefficient of variation was within 20%.

### Digital droplet PCR (ddPCR)

Plasma samples were pooled from the biobank aliquots and centrifuged for 14000xg for 20 min. The supernatant was carefully transferred for DNA isolations. Plasma DNA was extracted using the QIAamp MinElute ccfDNA Mini Kit (catalog no. 55204, Qiagen) following the manufacturer’s protocol. DNA was eluted and quantified using the Qubit® 2.0 Fluorometer (Life Technologies).

We used the Bio-Rad QX200 ddPCR system (Bio-Rad, Hercules, CA). OncobitTM PM, which consists of positive DNA controls and digital PCR assays targeting GNAQ209P, GNAQ209L, and GNA11209L mutations, was kindly provided by Oncobit AG (Switzerland). Control and primer sequences are proprietary to the company. Data was processed using QuantaSoft v.1.6 (Bio-Rad) and OncobitTM PM Analyzer.

For ddPCR analysis, 5 μL of cfDNA was added per reaction irrespective of the cfDNA concentration. Each run included a non-template control, and OncobitTM PM positive control for GNAQ.209P, GNAQ.209L, or GNA11.Q209L.

Droplets were generated using the Automatic Droplet generator from Bio-Rad. DNA was amplified according to the manufacturer’s instructions. The samples were held at 12 °C until further processing. Droplets were analyzed through a QX200 droplet reader (Bio-Rad). QuantaSoft analysis software (Bio-Rad) was used to acquire the data and Oncobit™ PM analyzer was used to analyze the data. Only samples that were analyzed successfully for all three mutations were included for analysis when the mutation status of the patient was unknown.

### Immunohistochemistry

 3 μm sections of metastases from uveal (*n* = 18) or cutaneous melanomas (*n* = 10) were used for cMET protein expression (anti-Met, ab216574, Abcam). The immunohistochemical (IHC) staining procedure was completely carried out on a BOND-RX (Leica, Germany) using a Bond Polymer Refine Detection kit (for DAB). The antigen retrieval was carried out in a pH 9.0 solution, and the primary cMET antibody dilution was 1/5000. The tissue sections were counterstained with hematoxylin. Slides were scanned at 20X using Olympus VS200. cMET protein expression was assessed for staining intensity and the percentage of stained tumor cells.

### Cell culture

Mel-202 (13012457, Sigma-Aldrich), 92–1 (13012458, Sigma-Aldrich), and A375 (88113005, Sigma-Aldrich) cells were purchased. WM35 cell line was kindly provided by Prof. Dr. Schittek, University Hospital Tübingen, Germany; SK Mel 28 cell line was provided by Prof. Dr. Ungerechts, University Hospital Heidelberg, Germany; HEPG2, and HEP3B cell lines were provided by Dr. Anna-Lena Scherr, NCT Heidelberg, Germany. HDM-93, HDM-117, HDM-396, and HDM-332 cells derived from skin metastasis of cutaneous melanoma patients with liver metastasis at the time of biopsy in-house. All cell lines were cultured in RPMI containing 10% FBS and 1% penicillin–streptomycin mixture (PenStrep), except for A375, WM35 and SK Mel-28, which were maintained in DMEM containing 10% FBS and 1% PenStrep. All cells were maintained with 5% CO2 at 37 °C, and the cell passaging was conducted using 0.25% trypsin + EDTA.

### Western blot analysis

The cells were lysed in ice-cold RIPA buffer [50 mM Tris–HCl (pH 8.0), 150 mM NaCl, 5 mM EDTA, 1% Triton X-100, 0.5% sodium deoxycholate and 0.1% SDS] containing protease inhibitors (Roche) to extract the total protein. Protein lysates were centrifuged at 12,000 × g for 15 min at 4 °C, and the protein concentration was determined using a bicinchoninic acid assay. Samples containing equal amounts of protein (30 µg per lane) were separated under reducing conditions using 4–20% SDS-PAGE, and the proteins transferred to PVDF membranes, followed by blocking with 5% milk in TBST at room temperature for one hour. Membranes were then incubated with primary antibodies (cMET (anti-Met, ab216574, Abcam), Melan-A (64718T, CST), beta-actin (3700T, CST)) overnight at 4°C. The primary antibodies were diluted in TBST with 5% milk (cMET: 1:1000; Melan-A: 1:1000; beta-actin: 1:1000). Following the incubation with the primary antibody, membranes were incubated with horseradish peroxidase-conjugated secondary antibodies diluted in TBS-T with 1% milk (donkey anti-mouse, catalog no. 715–036-151, Jackson Immuno Research), donkey anti-rabbit, catalog no. 711–036-152, Jackson Immuno Research). Membranes were visualized using the EZ-ECL chemiluminescent substrate reagent (catalog no. 34075, Thermo Fischer).

### The supernatant of melanoma cell lines

Melanoma cell lines were grown in 75 cm2 flasks until reaching 80–90% confluence. The cell culture medium was discarded and washed twice with RPMI/DMEM without FCS and then incubated for 24 h with RPMI/DMEM medium without FCS. After 24 h of incubation, the cell culture supernatant was collected under sterile conditions, centrifuged at 400xg for 10 min, and filtered through a membrane (0.2 µm, Millipore Corp.). The cell culture supernatant was stored at –80°C until use. Protein levels of cMET were determined using ELISA.

### Statistical analysis

Differences between continuous variables and groups were assessed using the Mann–Whitney U (MWU) test. Differences between categorical variables and ICI outcomes in mUM patients were analyzed using univariate logistic regression analysis. Baseline variables that achieved a significance level of p < 0.05 were included and further analyzed using multivariate analysis. Kaplan–Meier analysis and the Log-rank test were used for survival analysis, and the hazard ratio (HR) was determined using a Cox proportional hazard regression model. The cutoff for scMET was determined by ROC curve analysis using ICI response as an event. The scMET cutoff value (253 ng/mL) was then used to categorize the scMET concentrations into two groups (low: < 253 ng/mL; high: ≥ 253 ng/mL) for assessing PFS and OS in mUM patients. Baseline factors associated with OS in tebentafusp-treated patients were analyzed using univariate Cox regression analysis. The correlations between continuous variables were determined by Pearson’s coefficient. All statistical analyses were performed using SPSS version 26 (IBM), and the graphs for data presentation were created using GraphPad Prism version 8 (GraphPad Software, Inc., La Jolla, CA, USA). The bars and lines in the column graphs represent median values and 95% CI. All reported p-values are two-sided, and *p* < 0.05 was considered to indicate a statistically significant difference.

## Results

### Clinical and molecular characteristics of mUM and mCM patient cohorts

**Fig. 1:**
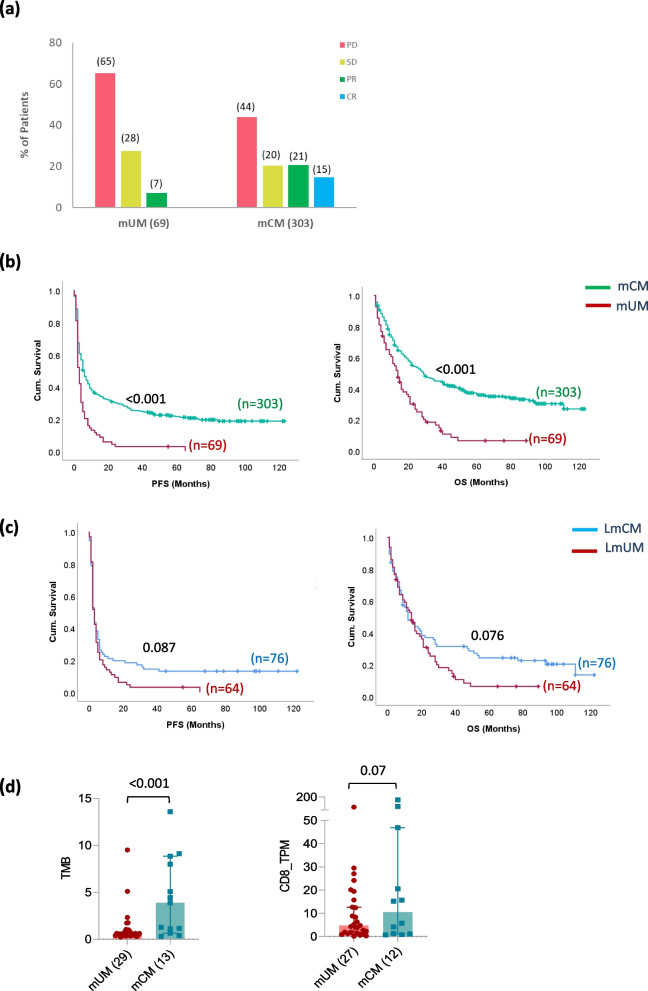
Clinical and molecular characteristics of our mUM and mCM patient cohorts: **a** Bar graphs indicating the percentage of tumor responses (PD vs. SD vs. PR vs. CR) in ICI-receiving mUM and mCM patients. **b** PFS and OS difference between mUM (red) and mCM (green) patients receiving ICI treatment. **c** The difference in PFS and OS between LmCM (blue) and LmUM (red) patients receiving ICI treatment. **d** Bar graphs showing the difference in tumor mutational burden and CD8 mRNA expression in patients included in the MASTER sequencing program. The bars and lines in the column graphs represent median values and 95% CI. *p* <.05 was considered to be statistically significant and was indicated above the respective graph

Between 2013 and 2024, 69 patients with mUM and 303 patients with mCM were treated with immune checkpoint blockers. Patients had a median age of 61 years at treatment start (range 17–94). Patients with mUM consisted of more females, received more frequently combined ipilimumab + nivolumab therapy, and had significantly more often liver metastases (Additional Table 1). Brain metastases were more frequent in mCM patients. Concerning clinical outcomes, the overall response rate was 7% for mUM and 36% for mCM, whereas the disease control rate was 35% for mUM and 56% for mCM (Fig. 1a). Median PFS was 6 months in mCM and 3 months in mUM, and median OS was 27 months and 14 months. Hence, mUM patients revealed a worse survival compared to mCM patients: PFS (*p* < 0.001, HR: 1.99, 95%CI: 1.5–2.6) and OS (*p* < 0.001, HR: 2.16, 95%CI: 1.6–2.9), as expected (Fig. 1b). As uveal melanomas metastasize predominantly to the liver, we then looked at the clinical outcomes based on the presence (LmCM/UM) or absence (nLmCM/UM) of liver metastasis at the time of ICI treatment start. Clinical characteristics in patients with liver metastases (LmCM/UM) were comparable, with the exception of brain metastases being more frequent in LmCM (Table 1). 76 (25%) of mCM patients had liver metastasis and PFS (p = 0.001, HR: 1.6, 95%CI: 1.2–2.1) and OS (*p* < 0.001, HR: 1.7, 95%CI: 1.3–2.4) was lower in LmCM patients compared to nLmCM patients (Additional Fig. 2). Moreover, 64 out of 69 mUM patients (93%) had liver metastasis. Here, the survival of the five patients with extrahepatic disease only was comparable to that of LmUM patients (Additional Fig. 2). However, LmUM patients tended to experience shorter PFS (*p* = 0.087; HR: 1.3, 95%CI: 0.9–1.9) and OS (*p* = 0.076; HR: 1.4, 95%CI: 0.9–2.0) when compared to LmCM patients (Fig. 1c). Hence, the lower efficacy of ICI cannot be explained purely by the hepatodominant metastasis pattern. As uveal melanomas are known to have a low tumor mutational burden (TMB), which explains ICI resistance in many tumor entities [26], we additionally analyzed the TMB and T cell infiltration in some of the patients included in the MASTER sequencing program. We noticed a lower TMB (*p* = 0.003) as well as a lower infiltration with CD8 + T cells (*p* = 0.07) in mUM patients compared to mCM patients (Fig. 1d).

**Table 1:** Clinical characteristics of ICI-receiving LmCM and LmUM patients

	LmCM (76)	LmUM (64)	*p*-value
Age (Years)			0.693
Median (Range)	55 (28–89)	60 (17–88)	
	n (%)	n (%)	
Gender			0.011
Male	48 (63)	26 (41)	
Female	28 (37)	38 (59)	
LDH			0.258
Normal	30 (39)	27 (42)	
Elevated	38 (50)	21 (33)	
Missing	8 (11)	16 (25)	
AST			0.088
Normal	42 (55)	22 (34)	
Elevated	25 (33)	26 (41)	
Missing	9 (12)	16 (25)	
ALT			0.088
Normal	42 (55)	22 (34)	
Elevated	25 (33)	26 (41)	
Missing	9 (12)	16 (25)	
Size of Liver Metastasis			0.422
M1a (< 3 cm)	41 (54)	25 (39)	
M1b (3-8 cm)	23 (30)	21 (33)	
M1c (> 8 cm)	5 (7)	6 (9)	
Missing	7 (9)	12 (19)	
Type of ICI			0.615
Pembro/Nivo	39 (51)	30 (47)	
IpiNivo	37 (49)	34 (53)	
Prior Systemic Treatment			0.608
Yes	35 (46)	26 (41)	
No	41 (54)	38 (59)	
Brain Metastasis			< 0.001
Yes	28 (37)	5 (8)	
No	48 (63)	59 (92)

### The M1 classification of liver metastases does not predict ICI outcomes in LmUM patients when metastatic lesions are less than 8 cm

**Fig. 2:**
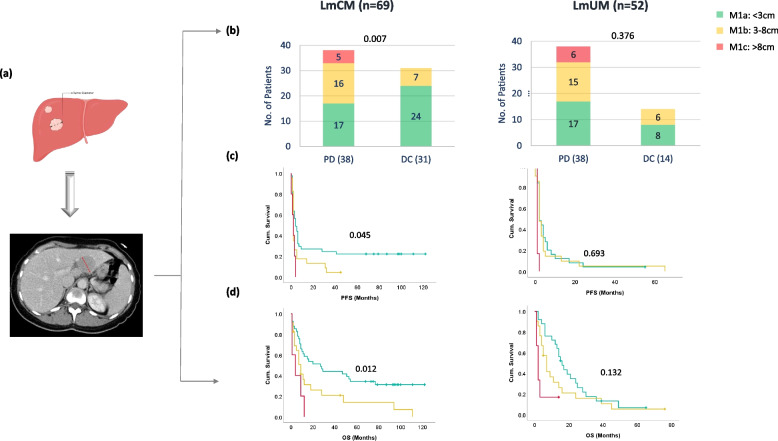
Correlation of liver metastasis size (M1 classification for uveal melanoma) with ICI outcomes in LmUM and LmCM patients: (a) Schematic and actual representation of liver metastasis size analysis. (b) Stacked column graphs indicating the number of patients with liver metastasis size of < 3 cm (M1a, green), 3-8 cm (M1b, yellow), and > 8 cm (M1c, red) in progressive disease (PD) or disease control (DC) in LmCM (left) and LmUM (right) patients. PFS (c) and OS (d) analysis of LmCM (left) and LmUM (right) patients on ICI treatment based on liver metastasis size < 3 cm (M1a, green), 3-8 cm (M1b, yellow), and > 8 cm (M1c, red). *P*-value was calculated using a log-rank test between the liver metastasis size groups: < 3 cm versus 3-8 cm, and are indicated above the respective graphs

To evaluate if the liver tumor burden influences clinical outcome, we used three different methods to measure the baseline tumor burden in the liver in patients with LmCM and LmUM. The pre-treatment radiological scans were available for evaluation in 69 LmCM (91%) and 52 (81%) LmUM patients. First, we used the size of the biggest metastasis as defined in the M1 classification for uveal melanoma (< 3 cm (M1a) versus 3-8cm (M1b) versus > 8 cm (M1c; Fig. 2a)), but for both entities.

Five LmCM patients (7%) and six LmUM patients (12%) had tumors larger than 8 cm in the liver at the time of ICI treatment start, and all of them had experienced disease progression within three months of ICI treatment. Comparing M1a and b metastasis, twenty-three LmCM patients with a metastasis size of 3-8cm in the liver (33%) had a significantly lower disease control response (*p* = 0.007), PFS (*p* = 0.045) and OS (*p* = 0.012) compared to forty-one (59%) patients with metastasis less than 3 cm (Fig. 2b, c, d). Interestingly, there was no difference based on M1 classification for LmUM patients for disease control response (0.376), PFS (*p* = 0.693), or OS (*p* = 0.132) (Fig. 2 b-d). Therefore, we tried a different radiological evaluation, namely measuring the total liver diameter (Additional Fig. 3). Here, the median liver diameter of all LmCM and LmUM patients was 22 cm (Range: 11-32cm), and no difference in the total liver diameter and treatment outcomes was observed in patients with LmCM or LmUM. Similarly, the estimation of total metastatic tumor volume in the liver once again revealed that all patients with tumor volume larger than 50% in the liver experienced disease progression. However, no significant differences were observed in the treatment outcomes when the tumor volume in the liver was less than 50% in both LmCM and LmUM patients (Additional Fig. 4).

### Routine baseline biomarkers correlated with ICI outcome in mUM patients

First of all, we tested if clinical characteristics and already known biomarkers from clinical routine blood assessments before treatment started correlated with ICI efficacy outcomes in mUM patients (Table 2). From the clinical features, patients who experienced rapid disease progression from primary melanoma to first metastasis were significantly more likely to be treatment-resistant and revealed a PD as the best response (median time interval 3.8 years, p = 0.018, HR: 4.3, 95%CI: 1,3–14.4). Significant biomarkers in the peripheral blood for ICI resistance were increased concentrations of AST (p = 0.005, HR: 10.6, 95%CI: 2.0–55), ALT (p = 0.027; HR: 5.1, 95%CI: 1.2–22), and LDH (p = 0.022, HR: 6.8, 95%CI: 1.3–35).

**Table 2:** Clinical characteristics of ICI-receiving mUM patients based on response

	PD (45)	DC (24)	*p*-value
Age (Years)			0.473
Median (Range)	61 (32–79)	62 (17–88)	
	n (%)	n (%)	
Gender			1.00
Male	20 (44)	10 (42)	
Female	25 (56)	14 (58)	
LDH			0.022
Normal	17 (38)	11 (46)	
Elevated	21 (47)	2 (8)	
Missing	7 (16)	11 (46)	
AST			0.003
Normal	13 (29)	11 (46)	
Elevated	25 (56)	2 (8)	
Missing	7 (16)	11 (46)	
ALT			0.027
Normal	15 (33)	10 (42)	
Elevated	23 (51)	3 (13)	
Missing	7 (16)	11 (46)	
Liver Metastasis			1.00
Yes	42 (93)	22 (92)	
No	3 (7)	2 (8)	
Size of Liver Metastasis			0.376
M1a (< 3 cm)	17 (38)	8 (33)	
M1b ((3-8 cm)	15 (33)	6 (25)	
M1c (> 8 cm)	6 (13)	0 (0)	
Missing	7 (16)	10 (42)	
Time from Primary to Metastasis			0.024
< 3.8 Years	23 (51)	5 (21)	
≥ 3.8 years	15 (33)	14 (58)	
Not applicable (Late diagnosis*)	7 (16)	5 (21)	
Type of ICI			0.125
Pembro/Nivo	23 (51)	7 (29)	
IpiNivo	22 (49)	17 (71)	
Prior Systemic Treatment			0.120
Yes	21 (47)	6 (25)	
No	24 (53)	18 (75)	
Brain Metastasis			0.652
Yes	4 (9)	1 (4)	
No	41 (91)	23 (96)	
Tumor Mutations			0.637
GNAQ	13 (29)	9 (38)	
GNA11	8 (18)	4 (17)	
GNAQ/GNA11 wt	2 (4)	0 (0)	
Missing	22 (49)	11 (46)	

^*^Not applicable: mUM patients whose first diagnosis was at stage III/IV

### Soluble cMET as a potential new biomarker for ICI outcome in mUM patients

**Fig. 3:**
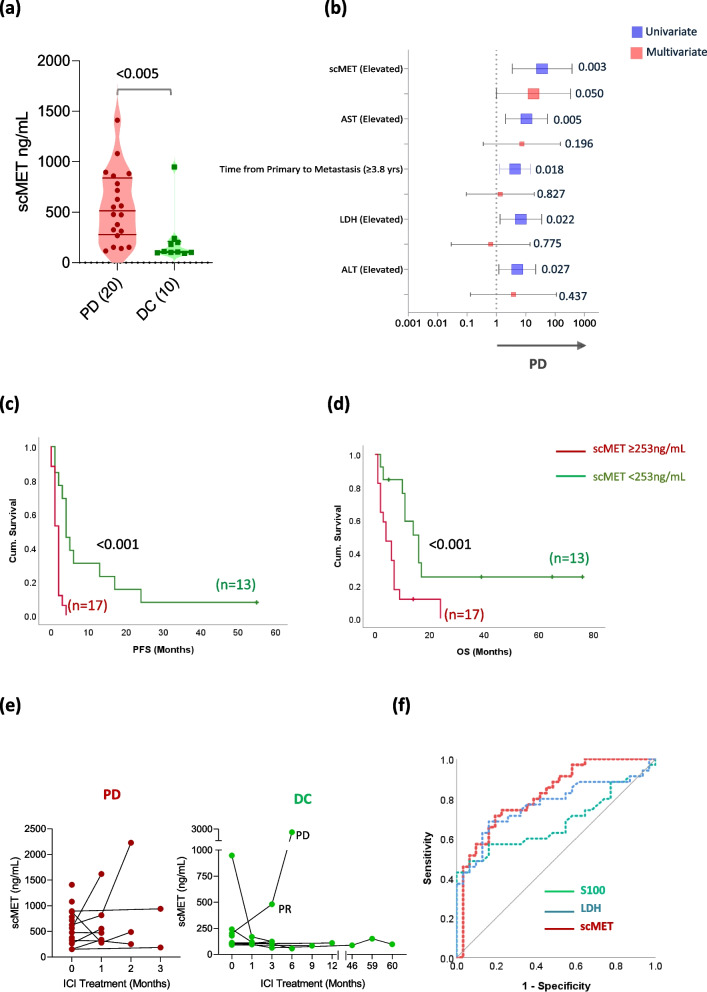
scMET as a potential biomarker for predicting ICI outcomes in mUM patients: **a** Violin plot showing the difference in pre-treatment scMET serum concentrations between mUM patients with progressive disease (PD) versus disease control (DC) as the best response to ICI treatment. **b** Forest plot showing potential baseline biomarkers associated with ICI outcome in uni (blue) and multivariate analysis (red). PFS (**c**) and OS (**d**) difference on ICI treatment in mUM patients with high (≥ 253 ng/mL, red) and low scMET (< 253 ng/mL, green) concentrations at pre-treatment. **e** Line graphs demonstrating the change in scMET concentrations during the course of ICI treatment in patients with PD (left) and DC (right). **f** Comparison of scMET, LDH, and S100 for predicting one-year overall survival in mUM patients in the validation cohort associated with high scMET levels also

cMET is a frequently overexpressed receptor tyrosine kinase in metastasis from uveal melanoma, activated by hepatocyte growth factor [17, 27]. In patients with metastatic uveal melanoma, it can be found as a soluble form (scMET) in the peripheral blood [19] but is not used in clinical routine as a tumor biomarker so far. We measured scMET in the patient sera at baseline and correlated it with ICI efficacy. We found a striking association between pre-treatment scMET concentrations and ICI response (*p* = 0.003, HR: 36, 95%CI: 3.5–373, Fig. 3a, b). When adjusted to the other potential baseline biomarkers, scMET remained the only significant factor associated with ICI outcome (*p* = 0.050; HR: 18.4, 95%CI: 1–338; Fig. 3b). Using ROC analysis, with response as an event, we found that patients with scMET concentrations above 253ng/mL were likely to experience a short PFS (*p* < 0.001; Fig. 3c) and OS (*p* < 0.001; Fig. 3d). Accordingly, on-treatment serum concentrations analyzed around 6 weeks after treatment initiation demonstrated the same trend. scMET concentrations were seen to be increasing or remained high in the on-treatment samples of non-responding patients, whereas a decrease in scMET concentrations was observed in disease-control patients (Fig. 3e). In a partially responding patient, increasing scMET was observed even before radiological progression was detected (patient marked with response in Fig. 3e, DC group). Furthermore, using a validation cohort with additional mUM patients including ninety-four mUM patients, where S100 and LDH were collected simultaneously, we could observe that scMET (*p* < 0.001; AUC: 0.818; 95%CI: 0.72–0.92) performed better than LDH (*p* < 0.001; AUC: 0.771; 95%CI: 0.66–0.88) and S100 (*p* = 0.012; AUC: 0.68; 95%CI: 0.54–0.81) for predicting one-year OS (Fig. 3f)). Here, a ROC cut-off of 255ng/mL was determined for scMET, similar to the cut-off defined by the ICI response, and it significantly predicted OS in this cohort (Additional Fig. 5a).

### Increased scMET concentrations are mUM specific and do not predict ICI outcomes in mCM patients

**Fig. 4:**
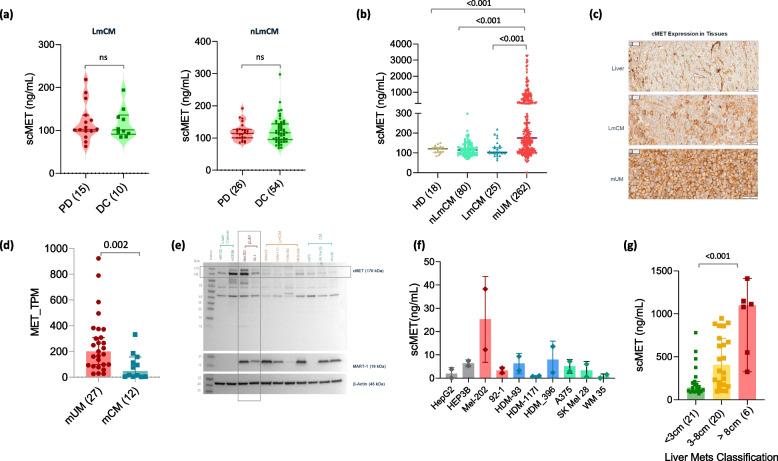
Increased scMET concentrations are mUM specific: **a** Violin plot showing the difference in pre-treatment scMET concentrations between patients with progressive disease (PD) and disease control (DC) as the best response to ICI therapy in LmCM (left) and nLmCM (right) patients on ICI treatment. **b** Violin plot showing the difference in scMET concentrations in the blood of healthy donors (HD, brown), nLmCM (green), LmCM (blue), and mUM (red) patients. **c** IHC expression of cMET in normal liver, LmCM, and LmUM tumor tissue samples. **d** Bar graphs showing the difference in cMET mRNA expression in tumor samples from mUM (red) and mCM (blue-green) patients in the MASTER cohort. **e** Western blot image showing the expression of cMET, and MART1 in UM (red), LmCM (orange), and CM (blue) and hepatocellular cancer (green) cell lines. Beta-actin was used as a loading control and is displayed at the bottom. **f** scMET concentrations in the cell culture supernatant from the respective cell lines. **g** Bar graphs showing the difference in scMET concentrations in mUM patient sera according to liver metastasis size (M1 classification)

To understand if scMET would be useful in predicting ICI response in mCM patients also, we then measured the pre-treatment concentrations of scMET in LmCM (*n* = 25) and nLmCM (*n* = 80). However, unlike in mUM patients, scMET concentration was not associated with treatment response (Fig. 4a). Interestingly, upon comparing all scMET concentrations between the cohorts, we could see that all samples (except for one nLmCM sample) with scMET concentrations of above 218 ng/mL were from mUM patients (Fig. 4b). Accordingly, a strong cMET expression was observed in tumor samples of mUM patients (Fig. 4c); however, in order to quantify the differential expression of cMET in mUM and mCM tumor samples, we used transcriptomic data from the MASTER cohort and observed that cMET gene expression was significantly upregulated in mUM compared to mCM tumors (Fig. 4d). As cMET expression is known in uveal melanoma cells [18, 28], we then examine if scMET derives from the tumor cells. We analyzed the cMET protein expression in UM (Mel-202, 92–1), CM (A375, SK Mel28, WM35), and LmCM cell lines (HDM-93, 117I, 396) with liver cancer cell lines as positive controls (HEPG2, HEP3B). In line with mUM patient tumor samples, we could see that cMET is over-expressed in Mel-202 UM cells (Fig. 4e), and accordingly, increased concentrations of scMET was observed in their supernatant (Fig. 4f). Furthermore, high scMET concentrations in patient sera correlated with larger liver metastasis in mUM patients (*p* < 0.001; Fig. 4g), high LDH (*p* < 0.001), elevated alkaline phosphatase (AP; *p* < 0.001) and elevated gamma-glutamyltransferase (GGT; *p* < 0.001). Interestingly, a reduced lymphocyte (*p* = 0.018) and eosinophil count (*p* = 0.009) in the peripheral blood were associated with high scMET levels also (Additional Fig. 5b).

### Combination of scMET, ctDNA, and LDH for monitoring early disease progression in mUM patients receiving immunotherapy with ICI

**Fig. 5:**
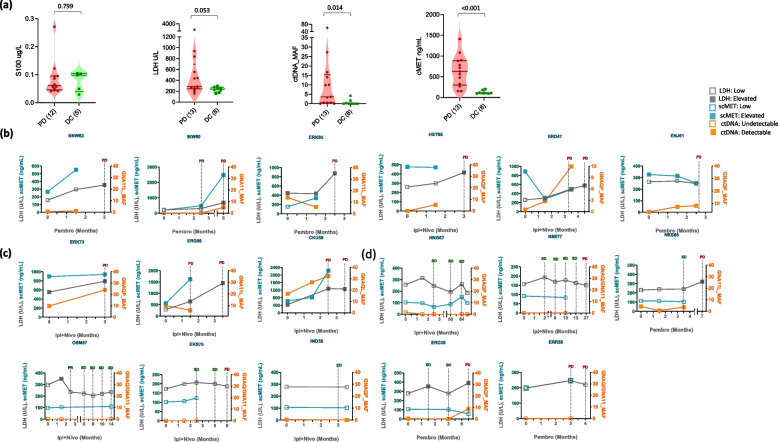
Simultaneous analysis of scMET, ctDNA, and LDH in ICI-treated mUM patients: **a** Violin plot showing the difference in pre-treatment S100, LDH, ctDNA, and scMET levels between mUM patients with PD and DC as the best response to ICI treatment. **b**, **c**, **d** Line graphs showing the changes in scMET (blue), LDH (grey), and ctDNA (orange) during the course of ICI treatments in individual mUM patients (b: early increase of scMET and/or ctDNA; c: parallel increase with LDH; d: patients with SD). The nearest radiological response to the time of analyzed samples was displayed on the graphs with the radiological outcomes: PD (red) or SD/PR (green), indicated on top of the dotted grey line

We then monitored scMET along with ctDNA (based on GNAQ and GNA11 mutations) and LDH in a small cohort of 23 mUM patients receiving ICIs (Fig. 5). ctDNA levels significantly correlated with LDH, with ctDNA being undetectable in patients with LDH less than 200U/L and detectable in most cases where LDH was above 320 U/L (Additional Fig. 5c). Before ICI treatment, ctDNA was detected in 12/23 (52%) mUM patients, and similar to scMET, we observed a significant correlation between ctDNA levels and ICI response at pre-treatment (p > 0.05; Fig. 5a). Notably, scMET and ctDNA performed better than LDH and S100 in predicting response to ICI treatment (Fig. 5a). In addition, on-treatment samples demonstrated a parallel increase of ctDNA along with scMET and LDH early on-treatment or at the time of staging in most of the patients with disease progression (Fig. 5b, c, d). In some patients who experienced disease progression, an increase in scMET and ctDNA was observed during early treatment at six weeks; however, LDH remained low/stable, demonstrating the potential of these markers for early detection of disease progression (Fig. 5b). In other patients, they increased in parallel (Fig. 5c). Whereas, in patients who experienced disease control, LDH and scMET remained low, and ctDNA was undetectable during the course of response (Fig. 5d).

### Combination of scMET, ctDNA, and LDH for monitoring early disease progression in mUM patients receiving immunotherapy with tebentafusp

**Fig. 6:**
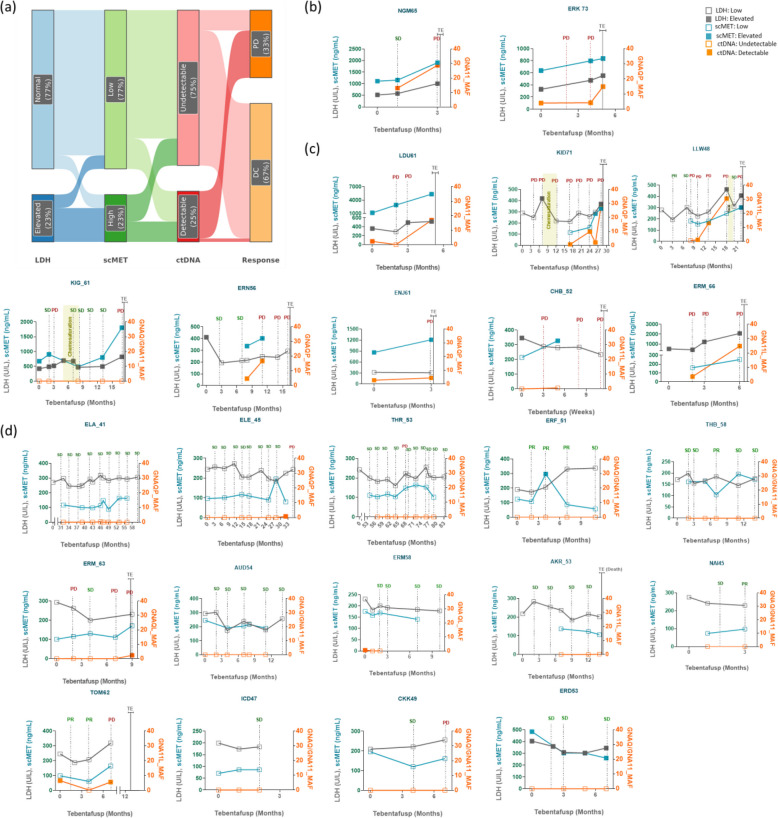
Simultaneous analysis of scMET, ctDNA, and LDH in tebentafusp-treated mUM patients: **a** Sankey diagram demonstrating the flow of all four markers: LDH, scMET, ctDNA, and radiological response collected simultaneously. **b**-**d** Line graphs showing the dynamics of scMET (blue), LDH (grey), and ctDNA (orange) during the course of tebentafusp treatment in individual mUM patients (b: parallel increase with LDH; c: early increase of scMET and/or ctDNA; d: patients with SD). The nearest radiological response to the time of analyzed samples was displayed on the graphs with the radiological outcomes: PD (red) or SD/PR (green), indicated on top of the dotted grey line. TE label on the graphs indicates the time when tebentafusp was ended

Tebentafusp is a new TCR-based immunotherapy for patients with mUM and an HLA-A*02:01 phenotype. One of the main clinical problems with tebentafusp therapy is the decision of when to treat beyond radiological progression and when to stop, as patients benefit despite PD in the staging [7, 15]. To understand if the above combination of biomarkers can be used for monitoring disease progression in mUM patients under tebentafusp, we prospectively analyzed scMET, ctDNA, and LDH in twenty-four patients receiving tebentafusp therapy (Fig. 6). This patient cohort revealed a median age of 66 years (range 47–49), and [13] 54% were male (Additional Table 2). Elevated levels of LDH were found in 8 (33%) patients at baseline. Among the pre-treatment blood samples, ctDNA was detectable in 5 of 14 (36%) patients, and scMET was elevated in 6 of 15 (40%) patients analyzed.

We analyzed the dynamics and flow of all four markers (LDH, scMET, ctDNA, and radiological response) collected simultaneously in these patients (Fig. 6a). We observed that among 33% (*n* = 20) of cases with tumor progression as a radiological response, in 20% (*n* = 4), scMET was low (below the cutoff 253 ng/mL), and ctDNA was undetectable, suggesting that these patients might benefit from treatment beyond radiological progression. In 25% (*n* = 5) of cases, scMET was high, and ctDNA was detectable (median MAF: 17%), suggesting that tebentafusp may no longer be effective in these patients. However, there are cases that show an increase in just one of the markers, leaving room for discussion. In 25% (*n* = 5), scMET was low, LDH was normal, but ctDNA was marginally detectable (median MAF: 2.3%). The slight increase in ctDNA might indicate an early progression. Likewise, in 20% (*n* = 4) of cases, scMET was low, but LDH was elevated (median: 438 U/L), and ctDNA was detectable (median MAF: 15%). Here, the elevation of two markers probably indicates disease progression. In two additional cases (10%), scMET was high, and ctDNA was undetectable, with LDH being normal in one (LDH: 288 U/L) and elevated in the other (LDH: 821 U/L), highlighting the necessity of combinational assessment of these markers in such cases. Similar to the ICI cohort, scMET and/or ctDNA increased before a rise in LDH and radiological disease progression was observed in many patients (Fig. 6c). In some patients, scMET was the earlier marker (e.g., LDU61, ERK73), and in some, ctDNA was more sensitive (LLW48, ERM66), demonstrating that the use of both markers would be of value to monitor early disease progression under tebentafusp therapy. Again, in 2 patients, the rise was parallel to LDH (Fig. 6b). In several patients with a stable clinical course, the markers were either undetectable or below the threshold of being positive (Fig. 6d). Of note, as we were measuring only the three most frequent GNAQ/11 mutations in the ctDNA analysis, some patients with non-detectable mutations might indeed be ctDNA positive but for a different mutation (e.g., KIG 61). This highlights the importance of prior knowledge of GNAQ/11 mutation status for ctDNA analysis using a targeted approach.

## Discussion

In this study, we demonstrate the significance of monitoring scMET concentrations in the blood of mUM patients for the early detection of tumor progression in ICI-receiving mUM patients. scMET outperforms LDH and S100 for predicting one-year OS in mUM patients. Using a validation set comprising LmCM and mCM patient samples and cell lines, we could show that increased concentrations of scMET are mUM specific, and the scMET concentrations do not indicate outcomes in ICI-receiving LmCM or mCM patients. In addition, monitoring scMET in combination with ctDNA revealed the potential to detect early tumor progression in mUM patients receiving ICI or tebentafusp treatment.

ICIs have limited efficacy in mUM patients, with response rates varying from 3 to 18% depending on treatment with anti-PD1 alone or combined with anti-CTLA4 [13, 29–31]. Therefore, a recommendation for their use must consider the balance of benefits and risks of immune-related adverse events (irAEs) associated with ICI therapy. Early markers for predicting ICI resistance are critical in this regard. Several prognostic biomarkers, such as LDH, CRP, and eosinophil count, have been reported previously, but they were not specifically predictive of ICI response [29]. In our study, LmUM patients with liver metastasis size (M1 classification) larger than 8 cm experienced rapid disease progression on ICIs; however, in our patient cohort, the M1 classification did not make a difference in LmUM patients when it comes to smaller liver metastasis (3cm versus 3-8cm), unlike in LmCM. Concerning blood biomarkers, we observed that the use of ICIs appeared to achieve higher response in a subset of mUM patients with low scMET, LDH, AST, and ALT. scMET concentrations of above 253ng/mL significantly determined mUM patients with poor PFS and OS under ICI therapy. In addition, we could also show that scMET outperforms the serum tumor markers S100 and LDH for predicting one-year OS using an additional mUM cohort. Increased concentrations of scMET were mUM specific as in LmCM and nLmCM patients only low concentrations were observed with no impact on ICI response noticed. This may be explained by the increased expression of cMET in mUM patients'tumor samples compared to mCM patient tumors. Although we did not analyze scMET concentrations in primary UM, a previous report showed an increased concentration of scMET in the blood of mUM patients compared to primary UM patients, implying that monitoring scMET concentration may help in the early detection of metastasis also [19]. In addition, by comparison of scMET to ctDNA and LDH measurements, we observed that scMET was the most sensitive baseline biomarker in mUM patients receiving ICI treatment. However, the on-treatment samples demonstrated the potential of scMET and ctDNA combinational assessment for avoiding the interpretation of false positive/negative results with any one of these markers. Together, these findings may help select patients who would benefit from ICI and help to detect treatment resistance early.

Tebentafusp is the first anti-cancer treatment to demonstrate an overall survival benefit in mUM patients, with a median overall survival of 21.6 months at a three-year follow-up compared to 16.9 months in patients in the control group [7]. This overall survival benefit is seen despite a low response rate of only 11%, with more than half of the patients revealing a PD as the best response. However, overall survival was better with tebentafusp compared to the control group, even in patients with PD as the best response, and more than half of the patients were treated beyond radiological disease progression in the phase III trial [7]. Here, an ongoing challenge is deciding how long to continue tebentafusp beyond progression and when to stop. Within the phase III trial, ctDNA was measured at baseline and at week 9, and from patients with measurable ctDNA at treatment start, more than 80% revealed a reduction independent of the RECIST response. This reduction in ctDNA correlated very well with patient survival [7, 22]. However, measuring ctDNA in clinical routine is challenging, and easier-to-detect biomarkers are warranted. Here, scMET, which can be detected by an ELISA, might have the potential for easy clinical use. In our tebentafusp patient cohort, we found that the combination of scMET with ctDNA especially helped to monitor disease progression reliably under therapy. Although the cohort was too small for conducting OS analysis, in our study, we observed a rise in ctDNA and/or scMET levels even before an increase in LDH and radiological progression in most of the patients under tebentafusp, suggesting their potential to monitor early disease progression. In addition, we also observed patients with radiological disease progression where all three markers, levels of scMET, ctDNA, and LDH, remained low/undetectable at radiological progression, suggesting that this decoupling of the markers may explain the survival benefit of tebentafusp beyond radiological progression. Besides, scMET can be highly valuable where prior knowledge of the mutation status in mUM patients is unavailable for ctDNA detection. Together, these data reveal scMET as a highly sensitive and reliable biomarker with the potential to predict early disease progression in mUM patients undergoing immunotherapy. Especially in combination with ctDNA and LDH, this is significantly more reliable than the radiology report, helping clinicians determine when to stop treatment beyond radiological progression. Given the ongoing challenges with LDH, S100, and radiological response to monitoring disease progression in tebentafusp-receiving mUM patients, a combinational assessment of scMET and ctDNA offers reliable, sensitive, and tumor-specific biomarkers to monitor early disease progression and may assist in therapy decision-making.

Several limitations that were associated with the present study warrant attention. Firstly, this was a retrospective cohort study with archived biobank patient material conducted at a single institution, and the number of enrolled patients was relatively small. In addition to the pre-treatment biomarkers described in our study, secondary mutations such as SF3B1 and BAP1 in mUM tumors have been previously shown to correlate with prognosis in patients and may be useful as predictive biomarkers [32]. However, we were unable to assess these mutations in this study. In addition, ctDNA analysis was based on mutations in GNAQ.Q209P, GNAQ.Q209L, and GNA11.Q209L alone, which might have influenced the sensitivity of this biomarker in this study. Therefore, further studies are needed to validate and explore the potential of scMET and ctDNA biomarker-based therapeutic interventions for mUM.

## Conclusion

Here, we present a clinically feasible approach of combined molecular assessment using scMET, ctDNA, and LDH during ICI and tebentafusp treatments to monitor early disease progression with increased confidence. This combinational approach may enable the prediction of early tumor progression before radiological imaging under ICI therapy and can help to decide how long to treat patients with tebentafusp beyond radiological progression. Although our findings should be interpreted within the study limitations and further evaluations are required to draw a definitive conclusion, we believe our study provides important insight into the appropriate selection/continuation of immunotherapy for mUM patients. To avoid differing results and for reliable applicability of these findings, the unification of the criteria for inclusion and processing of samples is necessary, which is possible with the help of multi-center collaboration to improve research for patients with this rare and aggressive tumor.

## Supplementary Information

Supplementary Material 1.

Supplementary Material 2.

## Data Availability

No datasets were generated or analysed during the current study.

## Abbreviations

UM: Uveal melanoma
CM: Cutaneous melanoma
mUM: Metastatic uveal melanoma
mCM: Metastatic cutaneous melanoma
LmUM: Liver metastasized uveal melanoma
LmCM: Liver metastasized cutaneous melanoma
nLmCM: Metastatic cutaneous melanoma without liver metastasis
ICIs: Immune checkpoint inhibitors
LDH: Lactate dehydrogenase
AST: Aspartate aminotransferase
ALT: Alanine transaminase
HGF: Hepatocyte growth factor
cMET: Hepatocyte growth factor receptor
ctDNA: Circulating tumor DNA
