# Supplementary material for: Monitoring soluble cMET and ctDNA in metastatic uveal melanoma patients to track early disease progression on immunotherapies
Source: J Exp Clin Cancer Res. 2025 Jul 19;44:213. doi: 10.1186/s13046-025-03451-2 (PMC12275281; doi:10.1186/s13046-025-03451-2)
Supplement: Supplementary file 1 — Supplementary Material 1. [file 13046_2025_3451_MOESM1_ESM.pdf]

Additional Figure 1

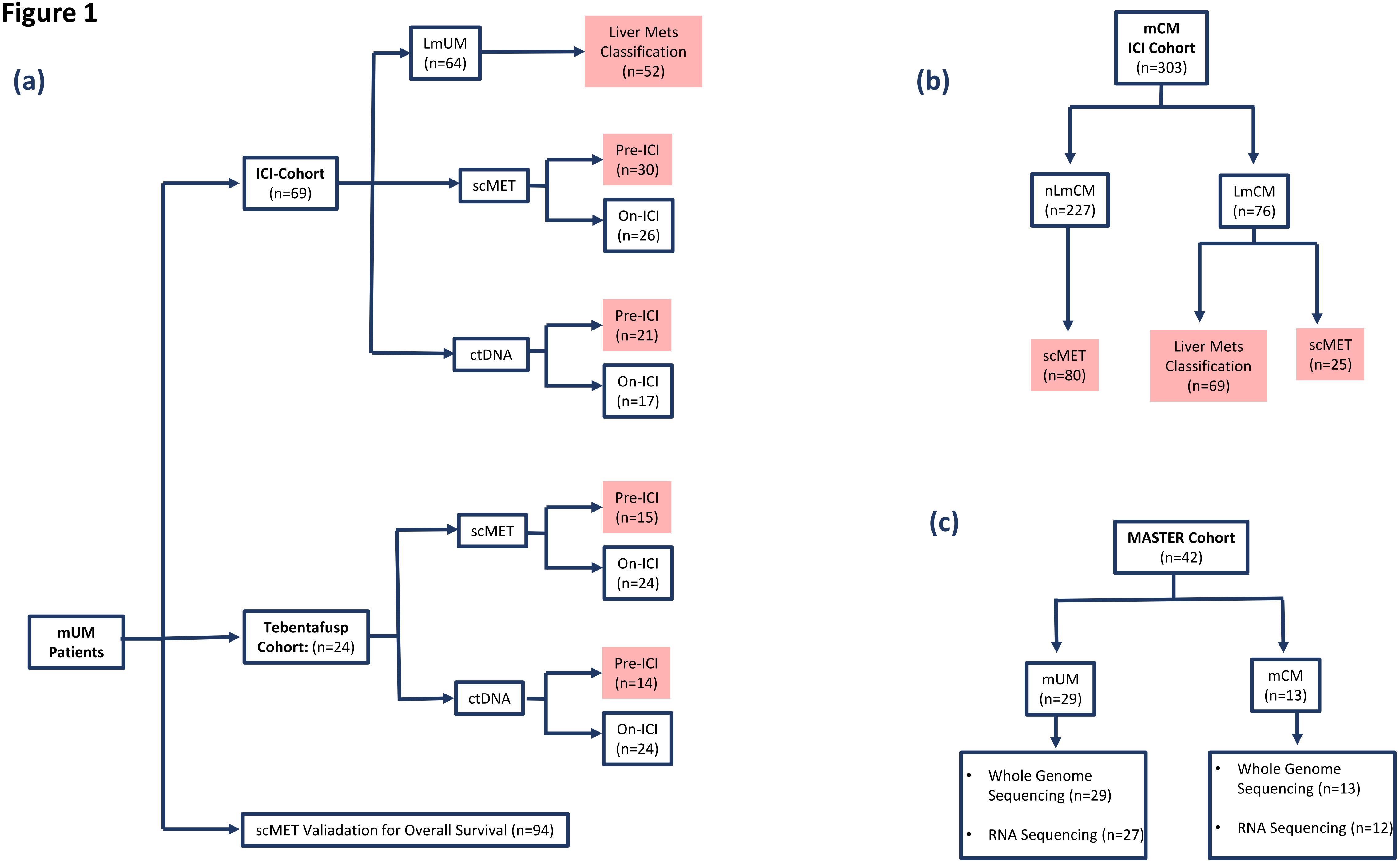

Additional Figure 1: Flow chart representations of the study cohorts. (a) mUM cohorts. (b) mCM cohorts. (c) MASTER cohort.

Additional Figure 2

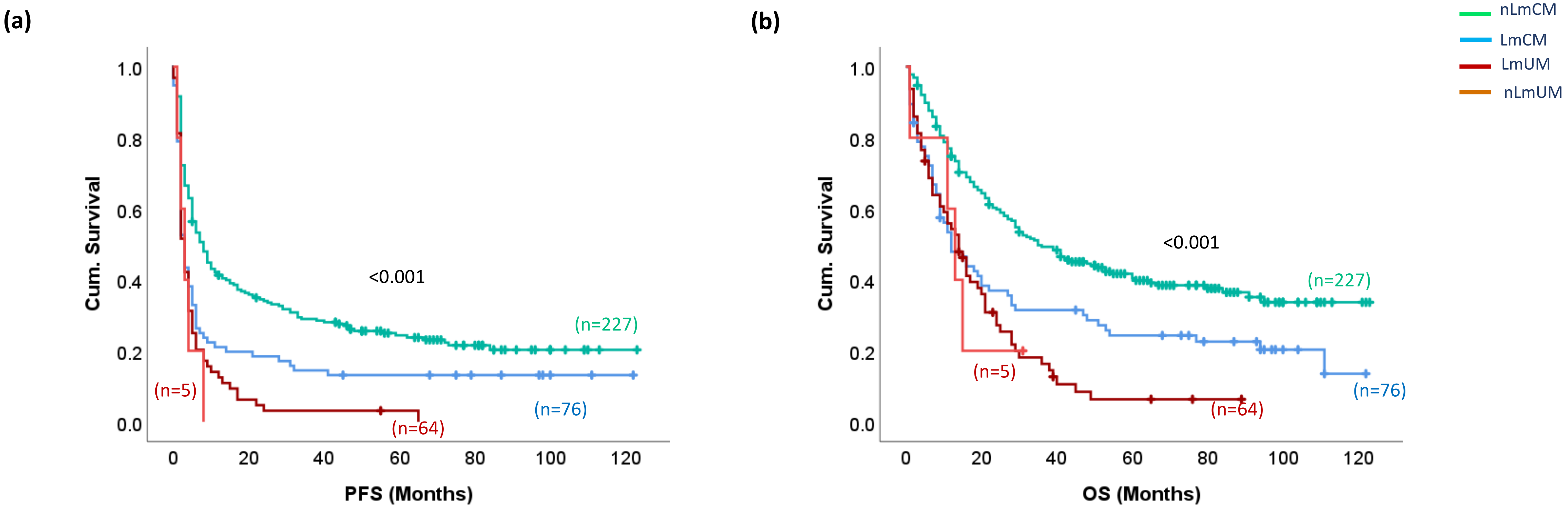

Additional Figure 2: (a) PFS and (b) OS difference between nLmUM (orange), LmUM (red), nLmCM (green), and LmCM (blue) patients receiving ICI treatment. $p < .05$ was considered to be statistically significant and was indicated above the respective graph.

Additional Figure 3

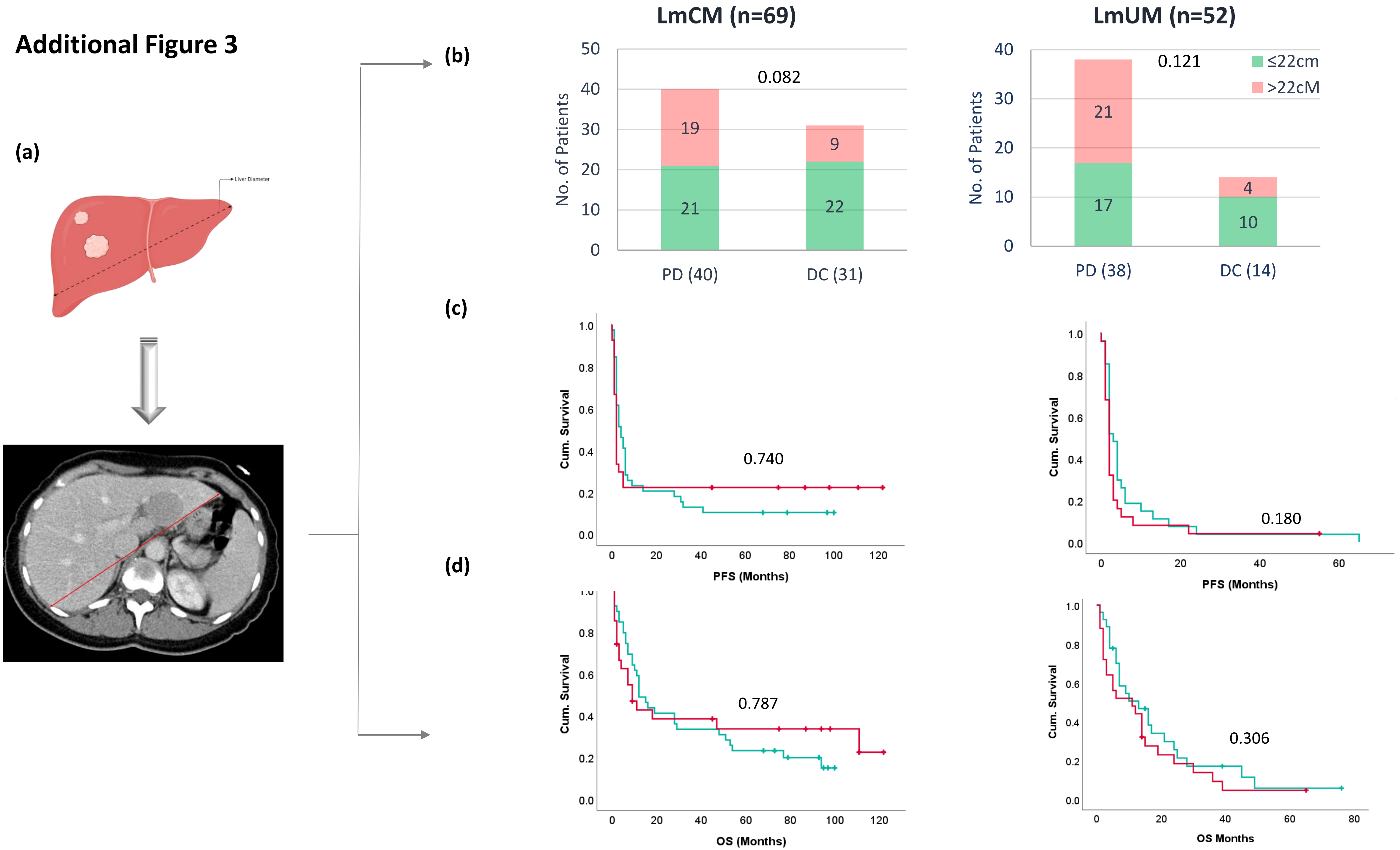

Additional Figure 3: (a) Schematic and actual representation of liver diameter analysis. (b) Stacked column graphs indicating the number of patients with liver diameter of ≤22cm (green), and >22cm (red) in progressive disease (PD) or disease control (DC) in LmCM (left) and LmUM (right) patients. PFS (c) and OS (d) analysis of LmCM (left) and LmUM (right) patients on ICI treatment based on liver diameter≤22cm (green), and >22cm (red). P-value was calculated using a log-rank test between the liver diameter groups: ≤22cm (green), and >22cm (red), and are indicated above the respective graphs.

Additional Figure 4

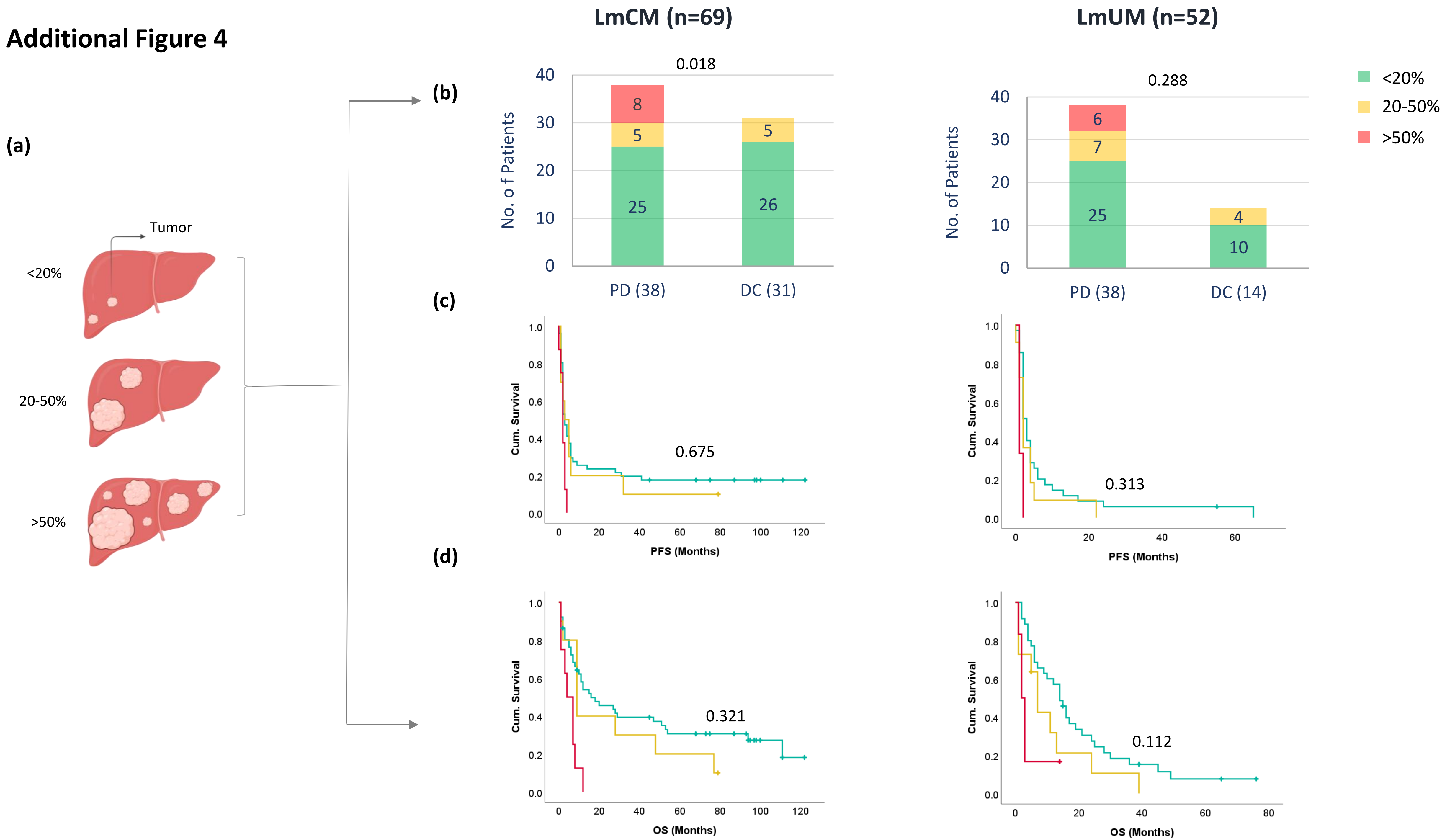

Additional Figure 4: (a) Schematic representation of estimation of metastatic tumor volume in the liver. (b) Stacked column graphs indicating the number of patients with metastatic tumor volume in the liver <20% (green), 20-50% (yellow), and >50% (red) in progressive disease (PD) or disease control (DC) in LmCM (left) and LmUM (right) patients. PFS (c) and OS (d) analysis of LmCM (left) and LmUM (right) patients on ICI treatment based on metastatic tumor volume in the liver <20% (green), 20-50% (yellow), and >50% (red). P-value was calculated using a log-rank test between the groups: <20% (green), 20-50% (yellow), and are indicated above the respective graphs.

Additional Figure 5

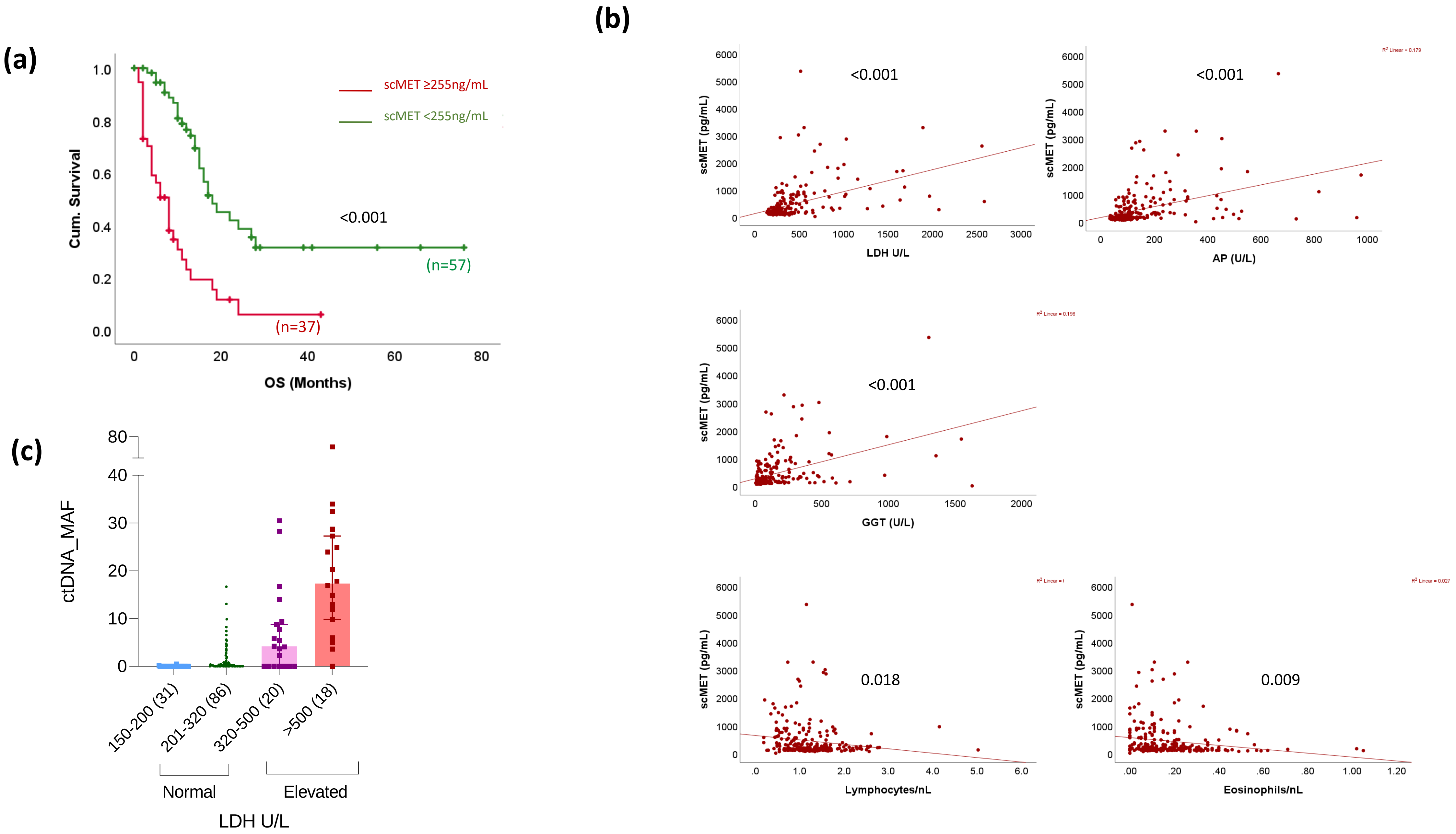

Additional Figure 5: (a) Difference in overall survival of mUM patients with high (≥255ng/mL, red) and low scMET (<255ng/mL, green) concentrations in the validation cohort. (b) scMET correlation with LDH, AP, GGT, Lymphocytes, and Eosinophils in the blood. (c) Bar graphs showing the differences in ctDNA MAF according to LDH levels in mUM patients.
