# Supplementary material for: Monitoring soluble cMET and ctDNA in metastatic uveal melanoma patients to track early disease progression on immunotherapies
Source: J Exp Clin Cancer Res. 2025 Jul 19;44:213. doi: 10.1186/s13046-025-03451-2 (PMC12275281; doi:10.1186/s13046-025-03451-2)
Supplement: Supplementary file 2 — Supplementary Material 2. [file 13046_2025_3451_MOESM2_ESM.docx]

| **Additional Table 1** |  |  |  |
| --- | --- | --- | --- |
| Clinical Characteristics of ICI-receiving mCM and mUM patients | | | |
|  | **mCM (303)** | **mUM (69)** | ***p-value*** |
|  |  |  |  |
| **Age (Years)** |  |  |  |
| Median (Range) | 61 (20-94) | 61 (17-88) | 0.824 |
|  |  |  |  |
|  | **n(%)** | **n(%)** |  |
| **Gender** |  |  | **0.022** |
| Male | 180 (59) | 30 (43) |  |
| Female | 123 (41) | 39 (57) |  |
|  |  |  |  |
| **LDH** |  |  | 0.114 |
| Normal | 179 (59) | 28 (41) |  |
| Elevated | 90 (30) | 23 (33) |  |
| Missing | 34 (11) | 18 (26) |  |
|  |  |  |  |
| **Type of ICI** |  |  | **0.033** |
| Pembro/Nivo | 175 (58) | 30 (43) |  |
| IpiNivo | 128 (42) | 39 (57) |  |
|  |  |  |  |
| **Prior Systemic Treatment** |  |  | 0.503 |
| Yes | 133 (44) | 27 (39) |  |
| No | 170 (56) | 42 (61) |  |
|  |  |  |  |
| **Liver Metastasis** |  |  | **<0.001** |
| Yes | 76 (25) | 64 (93) |  |
| No | 227 (75) | 5 (7) |  |
|  |  |  |  |
| **Brain Metastasis** |  |  | **<0.001** |
| Yes | 109 (36) | 5 (7) |  |
| No | 194 (64) | 64 (93) |  |

| **Additional Table 2** |  |
| --- | --- |
| Clinical Characteristics of Tebentafusp-Receiving mUM patients | |
|  | **Tebentafusp Cohort (n=24)** |
| **Age (Years)** |  |
| Median (Range) | 66 (47-79) |
|  | **n (%)** |
| **Gender** |  |
| Male | 13 (54) |
| Female | 11 (46) |
| **LDH** |  |
| Normal | 16 (67) |
| Elevated | 8 (33) |
| Missing |  |
| **Bone Mets** |  |
| Yes | 6 (25) |
| No | 18 (75) |
| **Prior Systemic Treatment** |  |
| Yes | 5 (21) |
| No | 19 (79) |
| **Any ICI before** |  |
| Yes | 4 (17) |
| No | 20 (83) |
